# Natural and Synthetic Clay Minerals in the Pharmaceutical and Biomedical Fields

**DOI:** 10.3390/pharmaceutics15051368

**Published:** 2023-04-29

**Authors:** Cristian Nomicisio, Marco Ruggeri, Eleonora Bianchi, Barbara Vigani, Caterina Valentino, Carola Aguzzi, Cesar Viseras, Silvia Rossi, Giuseppina Sandri

**Affiliations:** 1 Department of Drug Sciences, University of Pavia, Viale Taramelli 12, 27100 Pavia, Italy; 2 Department of Pharmacy and Pharmaceutical Technology, University of Granada, Cartuja Campus, 18071 Granada, Spain

**Keywords:** clay minerals, nanomaterials, drug delivery, tissue engineering, montmorillonite, halloysite, layered double hydroxides, zeolites

## Abstract

Clay minerals are historically among the most used materials with a wide variety of applications. In pharmaceutical and biomedical fields, their healing properties have always been known and used in pelotherapy and therefore attractive for their potential. In recent decades, the research has therefore focused on the systematic investigation of these properties. This review aims to describe the most relevant and recent uses of clays in the pharmaceutical and biomedical field, especially for drug delivery and tissue engineering purposes. Clay minerals, which are biocompatible and non-toxic materials, can act as carriers for active ingredients while controlling their release and increasing their bioavailability. Moreover, the combination of clays and polymers is useful as it can improve the mechanical and thermal properties of polymers, as well as induce cell adhesion and proliferation. Different types of clays, both of natural (such as montmorillonite and halloysite) and synthetic origin (layered double hydroxides and zeolites), were considered in order to compare them and to assess their advantages and different uses.

## 1. Introduction

Clay minerals, among the oldest materials of the planet, have long been used for several purposes. Moreover, since ancient times, it has been known that a number of clays possess antibacterial characteristics and can effectively treat wounds, soothe irritated skin, and stop bleeding [1].

Nowadays, clays are employed in various pharmaceutical and biomedical fields as enabling excipients to improve the technological and biopharmaceutical aspects of medicinal products since they provide unique functions due to their surface area, ion exchange capacity, layer charge, rheological and mechanical properties such as particle size, shape, and color [2,3].

Moreover, all of these properties make them suitable for applications in other fields, as shown in Figure 1, such as the treatment of wastewater [4], the adsorption of heavy metals [5], photocatalysis [6], packaging [7], and feed additives [8].

Several reports have been filed on the development of clay minerals in the pharmaceutical and biomedical fields [9,10,11,12]. For example, drug delivery systems containing clays are able to increase the solubility and permeability and control and prolong the drug release and its effectiveness, while reducing side effects and therefore increasing safety [13].

In the pharmaceutical and biomedical landscape, tissue engineering is a multidisciplinary field based on novel strategies for developing bio-inspired scaffolds to restore functional and structural characteristics of damaged tissue [14]. The development of polymer–clay nanocomposites is interesting in tissue engineering as clays enhance the mechanical properties of the polymers as well as favor cell adhesion and proliferation [15].

Numerous research papers and reviews regarding clay minerals and their role in drug delivery and tissue engineering have already been published, but none focus on the comparison of their performance depending on their origin and composition. Given this premise, this review focuses on the description of the most relevant clays and their most recent application in the pharmaceutical and biomedical field. Natural and synthetic clays are compared, and special attention is given to their biocompatibility and biopharmaceutical properties.

## 2. Structure of Clay Minerals

Clay minerals are hydrated aluminosilicates composed of aluminum and silicon oxides. They also contain numerous cations such as Na^+^, K^+^, Mg^2+^, Ca^2+^, and Fe^3+^. Structurally, the most common arrangement is presented by phyllosilicates, which are formed by stacked layers generally made up of continuous tetrahedral (T) and octahedral (O) sheets. These sheets are connected by hydrogen bonds that occur between the oxygen atoms of the tetrahedron and the hydroxyl groups of the octahedron. The formed structure can be assimilated to a 1 nm thick plate or disc. The lateral diameters of these plates range between 1 and 1000 nm, and the space between them is known as the interlayer space, which can be empty or filled with hydrated alkaline and alkaline earth cations. These components are altogether known as “structural units” and produce the clay particles. The overlapping of 5–10 parallel layers produces the so-called primary particle, whose thickness ranges between 8 and 10 nm and is independent of the interlayer distance. A group of randomly oriented particles forms an aggregate, as observed in Figure 2, whose dimensions are between 0.1 and 10 microns [16].

The arrangement of the layers or aggregates results in a variety of morphologies, including plates (montmorillonite, hectorite, and laponite) and fibers (sepiolite and palygorskite). Layers and sheets can also be arranged in hollow tubes, such as in the case of halloysite.

Depending on the nature of the interaction between the layers, different three-dimensional structures can be obtained, as the scheme reported in Figure 3 illustrates. The tetrahedrons, which are most typically composed of Al^3+^, Si^4+^, and Fe^3+^, are generally linked by basal oxygen atoms. The octahedrons, on the other hand, are connected to form hexagonal or pseudohexagonal layers by sharing edges. The apical oxygen of the tetrahedra allows the tetrahedral and octahedral structures to be attached, leading to different combinations [17]. For example, the 1:1 structure (T:O) is formed by the union of a tetrahedral and an octahedral sheet. This structure is extremely stable thanks to the hydrogen bonds established, which prevent the introduction of water molecules or other substances between the layers. This gives a basal interlayer distance of 7 Å. Kaolinite and halloysite are two types of clay belonging to this class. When the main layer is composed of two tetrahedral sheets wrapped around an octahedral sheet, as in the case of montmorillonite, the 2:1 structure is formed. In this case, the layer thickness is roughly 10 Å, while the basal distance varies between 9 and 15 Å, depending on the interlayer components. At the same time, the 2:1:1 category differs from the 2:1 structure because an octahedral layer occupies the interlayer space, creating a basal space of around 14 Å. Chlorites are included in this category [16].

In addition, hydrotalcite and its synthetic derivatives also possess a layered structure and are called layered double hydroxides (LDH) since they are formed by continuous cationic sheets which are separated by intercalated anions. However, their chemical composition differs from the previously mentioned clay minerals, as they do not belong to the aluminosilicate class. In fact, several different divalent and trivalent cations can be included in the structure. Mg^2+^ and Al^3+^ are the most widely used, but many other cations, such as Zn, Ni, Mn, Fe, Co, and Cr, are also used. Numerous anions can also be intercalated in the structure, both of inorganic or organic origin, as well as biomolecules [18].

Although it is the most common arrangement, the layered structure is not the only one which can be found. For example, zeolites are hydrated tectosilicates whose 3D structure is given by linked AlO_4_ and SiO_4_ tetrahedra. Several extra-framework cations, such as Na^+^, Li^+^, K^+^, Mg^2+^, and Ca^2+^, could also be present. The various structural accommodations of the tetrahedra allow the formation of a porous structure, which allows zeolites to exchange metal cations or host internal neutral guest molecules that can then be removed or replaced [19].

Chemical and structural properties impact the properties of clay materials. One of these is the charge of the layers comprising the clays. These may be negatively, positively, or essentially uncharged. This aspect, in turn, can affect the surface properties of these materials. For example, kaolinite and talc possess minimal layer charges and low cation exchange capacities (15–20 mEq/100 g). On the other hand, smectites such as montmorillonite present tetrahedral and octahedral substitutions as well as high ion exchange capacities (70–120 mEq/100 g) [20].

## 3. Current Use of Clay Minerals in the Pharmaceutical and Biomedical Field

Currently, clay minerals are used as excipients in pharmaceutical preparations. The most commonly used clay minerals belong to phyllosilicates such as kaolinite, palygorskite, sepiolite, smectites, and talc; tectosilicates such as zeolites; and smectites such as montmorillonite, saponite, and hectorite [20].

Given their consolidated use, official monographs are present in several pharmacopeias [21]. For example, fibrous clays such as palygorskite are recognized by the FDA as inactive components in oral solid dosage forms as they are considered nontoxic and non-irritant materials [22]. Moreover, clay minerals are used as excipients in pharmaceutical preparations to enhance their organoleptic characteristics, such as flavor and color. Mainly, they can be also used to improve the properties of the final product since they act as disintegrants due to their hydrophilicity, as lubricants, and as emulsifying, thickening, and anticaking agents due to their thixotropic and colloidal properties. In addition, their high adsorption capacity and specific surface area make them suitable as drug carriers [23,24]. Zeolites, as a part of tectosilicate clays, are also versatile materials for a wide range of applications in pharmaceutical technology. They are inert materials with low toxicity and their surface could be chemically modified to ensure their application as pharmaceutical excipients, even if these are not yet of consolidated use [25].

More recently, clay minerals have been proposed as an excipient for direct tableting—kaolinite proved to be easily compressible [26], while halloysite proved to give excellent mechanical properties and also to protect drugs against photodegradation, as in the case of nifedipine [27].

In addition, clay minerals could also act as active ingredients mainly as antacids or antidiarrhoeics [28] and these are related to their physico–chemical properties and their ionic composition [29].

Currently, in the biomedical field, clay minerals are modified to form nanocomposites. Two different types of nanocomposites are formed depending on the type of organic moieties considered: clay drug nanocomposites or clay polymer nanocomposites that could be further loaded with a drug. In this case, nanocomposites are carriers for extended-release drugs. The mechanism of drug loading and release and polymer interaction is affected by the functional groups and the physico–chemical properties of the drug and polymer and of the clay minerals considered. The loading is mainly related to the exchange capacity and therefore to the charge–charge interaction between the drug or polymer and the clay layers. The kinetic release is influenced by the type of ions and the ion concentration in the medium as well as by the pH. If a polymer is part of the nanocomposite, swelling or dissolution of the polymer could also occur.

Nanocomposites could also increase the bioavailability of poorly soluble drugs. Thus, the highly available surface area and the drug loading via molecule–clay interaction could increase the metastable solubility, resulting in higher drug absorption via physiological barriers, as has been assessed via an in vitro study on the Caco-2 cell line in the case of oxytetracyclin, isoniazide, and insulin. This finding is confirmed by in vivo studies as reported in the case of praziquantel and niclosamide.

Clay minerals are also able to exchange ions having a biological effect, and enhance tissue reparation or act as antimicrobials. Moreover, they are also currently used as fillers of a polymeric matrix (forming a nanocomposite mainly) to increase the mechanical properties of the scaffolds. In fact, the inclusion of a polymer to form a nanocomposite is beneficial for both drug delivery and tissue engineering applications: the polymer is able to assist drug delivery and the clay mineral is able to confer superior properties to the polymer matrix, giving higher mechanical and thermal properties and swelling capability. In tissue engineering, biodegradable and biocompatible polymers are the materials of choice for the development of 3D scaffolds to mimic the extracellular matrix of native tissue. Polymers, in fact, are ideal materials to manufacture 3D structures due to the hierarchical organization of their native tissues and their possession of biochemical cues to induce cell interaction, differentiation, faster tissue regeneration, and vascularization.

## 4. Natural Clays

The clays used in pharmaceutics are frequently natural substances, which are abundant and affordable, and only underwent little processing to ensure optimal purity and use. The geological landscape of the various deposits, however, may be responsible for some differences between these materials. Some minerals, such as kaolinite, talc, or sepiolite, show minor differences from the ideal composition; others, such as smectites, show isomorphic replacements as well as the many types of exchangeable cations that can enter the interlayer gaps [20]. Here, a few examples of pharmaceutical and biomedical applications of some naturally occurring clays are reported.

### 4.1. Montmorillonite

Montmorillonite (MMT), (general formula (Na, Ca)_0.33_(Al, Mg)_2_(Si_4_O_10_)(OH)_2_·nH_2_O), is a natural clay mineral belonging to the family of smectites. It possesses a 2:1 layered structure made up of an octahedral alumina sheet surrounded by two opposing tetrahedral silica sheets, as reported in Figure 4 [5].

The extra charge induced by the isomorphic replacement of cations inside the layers (for example, Al^3+^ replaced by Mg^2+^ in the octahedral sheet or Si^4+^ replaced by Al^3+^ in the tetrahedral lattice), causes the formation of an electrostatically charged structure. In fact, negative charges are present on the layers, which are compensated by alkali (Na^+^) or alkali earth (Ca^2+^, Mg^2+^) cations inside the galleries [31]. This characteristic also offers a broad number of interaction sites for drug molecules, particularly in the gap between layers, which may be expanded by intercalation. In fact, the interlayer spaces of MMT are hydrophilic and exhibit good swelling properties in the presence of water. The introduction of properly charged groups or molecules could improve the electrostatic stability of the clay [32].

The hydrophilicity of natural MMT, however, causes low compatibility with polymers, which prevents the wide application of this clay. As a result, several strategies have been used by researchers to produce modified MMT and improve its adsorption and ion exchange capabilities, therefore expanding its uses as a drug carrier and an antimicrobial [33]. MMT surface modification is critical to allow its spreading, dissociation, and exfoliation into individual layers. This reduces interlayer cohesive energy and creates a more favorable platelet–polymer interface [34].

MMT has a wide internal surface area, a high adsorption ability, a high cation exchange capacity (CEC), and low toxicity. MMT also has interesting properties such as strong thermal stability, a high Young’s modulus, high strength, and a low expansion coefficient, which are suitable for its use in biomedical fields [35].

#### 4.1.1. Drug Delivery Applications

MMT has been proposed as a drug delivery carrier to improve the bioavailability of poorly soluble drugs and to control drug release [36].

Nanocomposites based on MMT and different drug classes have been described in the literature. Moreover, in several works, MMT has been associated with polymers and mainly biopolymers to achieve higher drug loading and tune the interaction with biological substrates. Among the biopolymers, naturally occurring ones such as carrageenan, gelatin, chitosan, and alginate have been deeply investigated and other synthetic polymers such as polylactic-co-glycolic acid (PLGA), Eudragit PO, polyacrylamide, and polycaprolactone (PCL) were considered and different administration routes proposed.

In particular, as for the oral route, a nanocomposite based on MMT and PLGA loaded with dexamethasone (DEX) has been developed. The physico–chemical characterization confirmed the intercalation of both DEX and PLGA into MMT galleries [36]. MMT was also combined with i-carrageenan and gelatin, and ciprofloxacin was intercalated by means of an ion-exchange reaction. An ionic gelation reaction with Ca^2+^ ions allowed researchers to obtain beads from the loaded nanocomposites which were then subjected to freeze-drying. [37]. Furthermore, a nanocomposite based on MMT and chitosan and loaded with oxytetracycline was prepared using solid–liquid interaction. The system was able to increase drug bioavailability thanks to the internalization into enterocyte-like cells (Caco-2) [38]. In another work, MMT sodium alginate nanocomposites were loaded with curcumin and microbeads were prepared using different crosslinking agents. In particular, Ca^2+^ and Mg^2+^ conferred suitable properties for intestinal drug delivery to the systems [39].

Considering routes other than the oral one, Eudragit^®^PO and MMT nanocomposites intercalated with betaloxol hydrochloride were used to produce polymeric nanoparticles for ocular administration for the treatment of glaucoma. In an in vitro test, the drug delivery system was safe and well tolerated [40]. Moreover, an MMT/methylene blue nanocomposite was loaded into hydrogels based on polyacrylamide and sodium carboxymethyl cellulose for vaginal administration. The system allowed for a prolonged drug release and proved to be biocompatible in an in vitro cell model. MMT and methylene blue demonstrated a synergic antibacterial effect against *E. coli* [41].

#### 4.1.2. Tissue Engineering Applications

MMT has been also proposed as a functional component in tissue engineering to design different structures such as nanofibers, films, and sponges.

As for nanofibers, electrospinning was mostly employed, and both natural and synthetic polymers were considered. As examples, a polyurethane/MMT nanocomposite intercalated with chlorhexidine acetate (CA) and chitosan/pullulan/glycosaminoglycans/MMT intercalated with norfloxacin were designed to promote skin reparation and showed microbicidal effects in vitro [42,43].

Films based on bacterial cellulose and ionically modified MMT (Cu-MMT, Na-MMT, and Ca-MMT) were prepared. These were able to promote skin reparation and were effective against pathogens. Tissue regeneration, vascularization, and re-epithelialization were improved (Figure 5) [44].

As for bone regeneration, the freeze-drying process was used to produce a 3D chitosan-gelatin/nano-hydroxyapatite-MMT composite scaffold. MMT and hydroxyapatite had a fundamental role in mimicking both the nanoscale architecture and chemical content of the natural bone extracellular matrix. Moreover, they were able to enhance biomineralization and tune the mechanical behavior of the scaffold [45]. Analogously, films based on polyhydroxybutyrate/MMT were able to resemble the bone’s porous structure and its mechanical properties. The device was biocompatible in vitro and increased cell proliferation [46].

Moreover, a hydrogel based on silk fibroin and MMT was able to regenerate cartilage and bone at the same time. It also enhanced osteogenic development of bone marrow mesenchymal stem cells and stimulated osteochondral regeneration in vivo [47].

### 4.2. Halloysite

Halloysite (Al_2_Si_2_O_5_(OH)_4_ nH_2_O) is a dioctahedral 1:1 clay mineral found in soils, particularly in moist tropical and subtropical areas. Each deposit provides a peculiar purity grade, size, and hydration condition. Halloysite clay typically has a hollow tubular structure, even if alternative morphologies, such as platy and spherical, could be found. The outer surface of the halloysite tubes is made of siloxane (Si-O-Si) groups, while the interior surface is composed of a gibbsite-like array of aluminol (Al-OH) groups, as visible in Figure 6. This provides different charges on the surfaces of the nanoparticles over a pH range 2.5–8.5, which are positive on the inner surface thanks to the octahedral (Al(OH)_3_) sheet and negative on the outer one for the presence of SiO_2_ groups. However, in severely acidic circumstances, the outer surface of the halloysite becomes positively charged.

Halloysite nanotubes (HNT) are typically 1–2 μm in length, with the dimensions varying depending on the extraction site. Their outer and inner diameters are 50 to 100 nm and 10 to 50 nm, respectively, and they have an aspect ratio of approximately 20. The wall is made up of 10–15 bilayers with a gap of approximately 0.72 nm. The short dimensions of the tubes are appealing from a biological standpoint, since they are more suited for composites for sustained delivery of drugs. Moreover, two types of halloysite could be found depending on their hydration state, which affects the dimensions of the interlayer area dimensions: hydrated (halloysite—10 Å) and dehydrated (halloysite—7 Å).

The most intriguing element of halloysite clay is its different surface chemical composition, which enables selective modification and the creation of several attractive nanomaterials. Because of its unique morphology and charges, as well as its ease of modification by other materials and multiple methods, HNTs can be used to entrap a wide range of molecules, from negatively to positively charged substances and hydrophilic to hydrophobic compounds. Because of the presence of alumina, silica, hydrogens, and hydroxyls in the HNTs’ structure, many types of chemical bonds, including electrostatic, non-covalent, and covalent binding, might be formed.

These characteristics make HNTs promising carriers for the delivery of active ingredients including antimicrobial agents and enzymes. It has also been demonstrated that HNTs can improve the mechanical properties of several natural polymers, such as chitosan and alginate. Furthermore, HNTs are characterized by high biocompatibility and low toxicity [49,50,51].

#### 4.2.1. Drug Delivery Applications

As mentioned, HNTs have emerged as interesting candidates in drug delivery due to their capability to entrap a wide range of molecules.

Several studies have been performed to investigate the application of HNTs as drug delivery systems for oral applications. A nanocomposite based on isoniazid, a tuberculostatic agent belonging to class III of the BCS, and HNTs was designed to improve oral drug bioavailability. Permeability studies revealed that isoniazid transport across Caco-2 cellular membranes was improved, and the nanocomposite was efficiently internalized by the cells [52]. Similarly, naproxen-loaded HNT and ethylcellulose/hydroxypropylmethylcellulose blends were processed by spray drying (Figure 7), and a nanocomposite was obtained by ionic interaction [53].

HNTs have also been proposed for other applications, such as cancer, vaginal, and to enhance blood–brain barrier permeation.

HNTs coated with chitosan have been developed to enhance the release of curcumin in human breast cancer cell lines. The system allowed a slow and controlled release of curcumin [54].

HNTs have also been designed as nanocarriers able to permeate the blood–brain barrier and deliver drugs effectively for a prolonged period of time. The HNT loaded with rhodamine isothiocyanate and ionomycine, as a positive stimulus for Ca^2+^ response, exhibited a sustained and gradual drug delivery mechanism in brain microvascular endothelial cells, preventing permeation through the blood–brain barrier [55].

Furthermore, cyclodextrin and HNTs were used for the treatment of vaginal or buccal candidiasis as a carrier for clotrimazole prolonged release. The nanocarrier was produced by functionalizing the surface of the HNT with cyclodextrin moieties through microwave irradiation to facilitate interaction with the drug. In order to use the nanomaterial for local administration, several ammonium groups were grafted onto the hybrid system to provide mucoadhesion [56].

#### 4.2.2. Tissue Engineering Applications

Several works report the interaction between poly(lactic acid) (PLA) and HNTs by hydrogen bond formation between the carboxyl functional group of PLA with the hydroxylated internal and edge surface groups of HNTs. Van der Waals attractions between the lactic acid and HNTs, as well as hydrogen bonds, are responsible for the formation of the bonding mechanism in the halloysite nanotube–PLA nanocomposite [57]. This leads to increased thermal and mechanical properties of the nanocomposite [58,59,60] which are useful for the development of scaffolds for tissue engineering purposes.

For instance, scaffolds made of PLA/HNT and manufactured by foam injection molding process were developed. PLA and HNTs were first combined on a twin-screw extruder using the melt mixing technique. Then, foam injection molding was used to create tensile samples from neat PLA and PLA/HNT pellets. The mechanical properties of both the solid and foamed PLA/HNT were enhanced by the inclusion of HNTs in the matrix. Moreover, PLA/HNT scaffolds were also characterized by greater cell viability [61].

Chitosan is also deeply studied as a polymeric support for HNTs. In a work of ours, a nanocomposite was developed using HNTs and chitosan oligosaccharides as a powder to promote chronic wound healing. Hydrogen and electrostatic bonds were responsible for oligosaccharide–HNT interaction. Moreover, the nanocomposite proved to be biocompatible with normal human dermal fibroblasts, and their proliferation and migration were increased. Compared to pristine HNTs or chitosan oligosaccharides, the HNT/chitosan oligosaccharide improved skin reepithelization in an in vivo wound model [62].

Similarly, the electrospinning technique was used to manufacture chitosan/poly (vinyl alcohol) hydrogel nanofibers reinforced with HNTs. The tensile strength of the nanofibers enriched with HNTs increased to 2.4 and 3.5-fold more than bulk nanofibers. Moreover, HNTs increased the scaffold’s hydrophilicity, consequently promoting the adhesion of fibroblast cells [63].

In another work, a nanocomposite film based on dialdehyde–corn starch/gelatin/bacterial nano-cellulose was developed and loaded with HNTs combined with zinc oxide. The film greatly increased the proliferation of NIH-3T3 fibroblast cells. Moreover, HNTs and zinc oxide had a synergic antibacterial effect, leading to an inhibition of the growth of *E. coli* and *S. aureus* [64]. HNTs were also combined with Au nanoparticles and loaded into a chitin hydrogel. The system demonstrated high antibacterial and hemostatic activities (Figure 8), as well as wound healing capabilities with low cytotoxicity [65].

A hydrogel based on sodium alginate and collagen was also developed and HNT clindamycin phosphate nanocomposite was loaded for bone regeneration. Antibacterial tests in vitro confirmed the strong activity of HNT-loaded hydrogel. Moreover, the potential use in bone regeneration applications of the hydrogels was demonstrated [66].

Another method widely used for the design of scaffolds enriched with HNTs is freeze-drying. In particular, a polycaprolactone-polyethylene glycol-polycaprolactone/gelatin tripolymer was used to produce 3D nanocomposite scaffolds. Biocompatible nanohydroxyapatite (nHA), iron oxide nanoparticle (Fe_3_O_4_), and HNT powders were mixed in the polymer matrix to obtain a scaffold with great mechanical properties and osteogenic activity [67].

### 4.3. Sepiolite and Palygorskite

Fibrous clays, such as sepiolite (ideal formula: Si_12_O_30_Mg_8_(OH)_4_(OH_2_)_4_·8H_2_O) and palygorskite (ideal formula: Si_8_Mg_5_O_20_(OH)_2_(OH_2_)_4_·4H_2_O), are natural non-planar hydrous phyllosilicates with a 1D nanofibrous shape. Fibrous clays have always been extensively employed in the pharmaceutical and biomedical fields, despite being scarcer in geological relative distribution than other layered clays such as kaolinite and montmorillonite. Just like other layered clays, they possess a continuous two-dimensional tetrahedral sheet. However, they differ due to the lack of continuous octahedral sheets, which can be thought of as 2:1 phyllosilicate ribbons. This occurs because of the periodic inversion of apical oxygens in the continuous tetrahedral sheet, which causes the linkage of the ribbons. This inversion occurs every six atoms of Si (three tetrahedral chains) for sepiolite and every four atoms of Si (two tetrahedral chains) for palygorskite, allowing the formation of parallel structural tunnels, named channels, along the fibrous structure, as visible from Figure 9. Due to a larger density of silanol groups covering the external surface, fibrous clays could have better performances as nanofillers or nanocarriers [68].

Palygorskite, also known as attapulgite, has a similar architecture to sepiolite, but the channel dimension of sepiolite is mildly wider (10.6 Å × 3.7 Å) than that of palygorskite (6.4 Å × 3.7 Å). The composition and appearance of these fibrous clays can vary greatly depending on the natural deposit. The length of these fibers typically ranges between 1 and 2 μm, although in some situations they can be significantly longer [69].

Fibrous clays are characterized by remarkable surface features such as large specific surface area, strong ion exchange capacity, and numerous surface adsorption sites due to their high aspect ratio, rich surface silanol groups, and natural structural channels. These properties cause these micron-sized needle-like fibrous clays to form hydrogen bonds and assemble with a polymer matrix, making them useful for drug formulations [70]. The physico–chemical properties of fibrous clays and their differences from layered clays are reported in Table 1 and visible in the scheme reported in Figure 10.

The fibrous-like structure, contrary to platelet-like structures such as MMT, is able to easily diffuse into the polymer matrix and to avoid flocculation within the polymeric network, providing a strong mechanical strengthening effect. However, these clays lack affinity for hydrophobic organic polymers, and in those cases, a surface modification to ensure uniform dispersion into the polymer matrix is needed [71].

#### 4.3.1. Drug Delivery Applications of Sepiolite

Sepiolite was used to prepare a nanocomposite with oxaprozin, a poorly water-soluble anti-inflammatory drug, due to its higher adsorption capacity. Preliminary tests demonstrated that oxaprozin’s dissolution efficiency increased when it was entrapped in the nano clay due to the complexation or extremely fine dispersion of the drug in the clay structure [72]. A similar approach was used with hydrochlorothiazide: its interaction with sepiolite resulted in a synergic enhancement of drug dissolution properties, which increased when compared to the pristine drug [73].

A new material based on MMT and sepiolite clays, combined with two biopolymers (carboxymethylcellulose and zein), was developed to design drug-loaded nanocomposite films for topical administration and loaded with neomycin. The antibacterial properties of the system were assessed, and the results demonstrated its controlled release over time [74]. A combination of sepiolite and MMT was also suggested as a promising low-cost nanocarrier for the development of praziquantel delivery systems. The drug was easily intercalated into the MMT interlayer space and sepiolite channels, bypassing its poor water-solubility [75].

Furthermore, poly(vinyl alcohol)/soy protein isolate nanofiber mats were electrospun and employed as ketoprofen drug carriers. Sepiolite was employed to control drug release from the nanofiber mats. The study revealed that sepiolite strongly affected ketoprofen release. Moreover, the mechanical strength was enhanced with sepiolite addition to the nanofibers, enhancing their handling and stability [76].

#### 4.3.2. Tissue Engineering Applications of Sepiolite

Numerous scaffolds for tissue engineering applications were developed incorporating sepiolite. Freeze-drying was used to design porous poly(vinyl alcohol)/sepiolite nanocomposite foams, which were further thermally crosslinked with poly (acrylic acid). Sepiolite additives were found to increase the mechanical properties of the poly(vinyl alcohol) due to the effective dispersion of sepiolite into the polymeric matrix. Nevertheless, the material demonstrated potential to be further developed as a bone scaffold [77].

Alternatively, a casting method was used to develop chitosan–sepiolite nanocomposite films. The increase in the system’s wettability underlined the clay’s water-holding capability, resulting in a moist environment that is crucial for wound healing. Sepiolite also improved blood clotting ability and antibacterial activity against both Gram-positive and -negative microorganisms, showing good in vitro biocompatibility towards fibroblasts [78].

A comparison between MMT and sepiolite was carried out loading the different clays onto a chitosan/silk peptide film. The dispersion of sepiolite in the matrix resulted in an increased wettability of the system. Moreover, the improvement of the mechanical properties of the chitosan/silk peptide matrix was associated with a better dispersion of the sepiolite in the polymeric matrix with respect to MMT [79].

The solution casting approach was also used to create pH-sensitive, biodegradable, and antibacterial hydrogels from pectin and polyvinylpyrrolidone enriched with sepiolite clay. The clay concentration in the polymeric mixture influenced the hydrogels’ swelling. Moreover, the hydrogels were cytocompatible with fibroblasts when tested in vitro [80].

#### 4.3.3. Drug Delivery Applications of Palygorskite

Palygorskite (PAL) has been reported as a material able to enhance the bioavailability of drugs and control their release. pH-responsive systems and gels based on PAL have been exploited in drug delivery applications.

A pH-responsive system for rifampicin release based on PAL was used to avoid the major adverse effects associated with its long-term use. The interaction between the drug and the clay surface sites occurred via the formation of hydrogen bonds. In addition, the palygorskite surface was pH-dependent, allowing for higher drug bioavailability at pH levels that mimic the gastrointestinal environment. As a result, the drug can be administered in lower dosages during the day, boosting therapeutic efficacy while decreasing toxicity [81].

A pH-responsive drug delivery system for isoniazid was also designed using PAL as a nanocarrier [82]. The equilibrium and thermodynamic factors of isoniazid adsorption onto a PAL were studied. The mechanism involved drug adsorption on activated palygorskite sites and a mild precipitation phase of drug molecules over the adsorbed monolayer. The nanohybrid formed spontaneously, and the reaction proved to be exothermic and exoentropic [83].

In another work, a nanocomposite based on PAL and tea tree oil (TTO) was designed for topical acne treatment. TTO release was extended, and skin sebum absorbability was enhanced. Moreover, the nanocomposite highlighted clinical therapeutic potential in the treatment of facial acne by rapidly decreasing inflammatory lesions, regulating skin sebum overproduction, and restoring barrier properties [84].

PAL and/or bentonite were also used as gelling agents for topical administration of curcumin [85] or diclofenac [86] nanocrystals. The wet ball media milling process was used to prepare a curcumin/diclofenac nanosuspension which was then incorporated into clay-based hydrogels using a homogenization process. PAL played an important role in the rheological behavior and, as a result, in drug nanocrystals release.

In another work, chlorhexidine diacetate was loaded into MMT or PAL for antibacterial applications. The results revealed that PAL released the drug slower than MMT, presumably due to more persistent interactions between the drug and the clay [87].

As a result of their synergistic effects, hybrid systems of PAL and chitosan showed enhanced performances in biological applications. Chitosan beads crosslinked with sodium tripolyphosphate were compared with PAL/chitosan (Pal/CS) beads both loaded with diclofenac (DC). The addition of PAL to the chitosan matrix led to a considerable decrease in the overall amount of DC released [88].

#### 4.3.4. Tissue Engineering Applications of Palygorskite

Different systems based on PAL have been designed for tissue engineering.

A composite based on PAL/neomicin and polyvinyl alcohol was prepared via tape casting for wound healing applications [89]. In another work, conventional free radical polymerization was used to produce a 3D network structure poly(acrylic acid)/PAL microgel. The inclusion of microgels in a PVA hydrogel showed remarkable mechanical strength useful in tissue engineering [90].

Moreover, a synthesized active silica nanorod from natural PAL has been developed and compared to pristine PAL as a nano-filler of chitosan/polyvinylpyrrolidone films. The PAL derivative had a higher dispersibility than PAL in the vehicle, and its addition greatly enhanced the mechanical properties of the films [91].

## 5. Synthetic Clays

Despite the large number of natural clays, they possess certain significant drawbacks compared to synthetic clays. Among these, it is worth mentioning the chemical diversity depending on the extraction origin, which could also cause variations in color and textural qualities. Moreover, crystallographic flaws connected to the genesis process and the location of the deposit, as well as the impurities and pollutants found, could prevent their application. So, their use in scientific fields that need a high level of chemical control, such as the biomedical field, could be limited. In some cases, chemical processes such as purification treatments could enhance the physico–chemical properties of natural clays [92]. Nevertheless, the synthesis of clays started to obtain materials with controlled characteristics due to the ease of the synthetic process. Here, some examples of the use of synthetic clays are reported, focusing on some of the most innovative types of clays in drug delivery and tissue engineering.

### 5.1. Synthesis Strategies of Clay Minerals

Different synthesis methods are reported in the literature that cover two strategies: natural clay mineral derivatization and total synthesis.

In the case of derivatization, the surface modifications of pre-existing natural forms allow for improvement of the loading propensity of different moieties, and also interaction with polymeric chains. In the second case, total synthesis is performed to obtain high-purity compounds. The methods mimic the natural conditions for clay generation [93,94] and shorten the natural processes which usually occur over long periods of time. The hydrothermal method is performed using stainless steel autoclaves with autogenous pressure [92,95] and clay minerals with tunable physico–chemical properties are obtained by controlling the nature of the precursors, temperature, time, and pH. Microwave-assisted synthesis is a valid alternative to the hydrothermal approach since it involves a lower temperature and shorter time with higher sustainability [96]. LDH and zeolites are usually prepared using both hydrothermal and microwave-assisted methods [97,98]. Coprecipitation is a further method that is based on the precipitation of salts in alkaline solutions or via anion exchange. Both of these processes are fast and do not require high temperatures [99]. Furthermore, innovative synthesis strategies, which are much more sustainable, are also under development and the solvent-free approach is reported for the synthesis of zeolites which leads to the formation of hierarchical porous structures with improved characteristics [100].

### 5.2. Laponite

Laponite is a synthetic trioctahedral hectorite-like clay mineral. It belongs to the smectite clay group and has excellent colloidal characteristics. Laponite is composed of octahedral layers of magnesium oxide interposed between two parallel trioctahedral layers with the formula (Na^+0.7^[(Si_8_Mg_5.5_Li_0.3_)O_20_(OH)_4_]^−0.7^. Synthetic production methods have resulted in disk-shaped crystals of 1 nm thickness and diameters of 25–30 nm. Laponite is negatively charged as a result of the isomorphic replacement of magnesium ions (Mg^2+^) with lithium ions (Li^+^). The net negative charge on the surface is balanced by cations such as sodium ions (Na^+^) [101].

Depending on the pH of the surrounding environment, the exposed hydroxyl groups on the edges of laponite crystals can be protonated and display a positive or less negative charge. Laponite generates transparent colloidal dispersions in water at low quantities. This is explained by the sodium-cation-containing electrical double layers that surround each crystal, which produce electrostatic repulsion forces between them. The addition of ions or polar molecules to the water may increase the attractive forces, resulting in the development of gels. The weak positive charges around the edges of laponite crystals might interact directly with the negative charges on the surface of other crystals, producing the “house of cards” self-assembling gel, as shown in Figure 11 [102].

A wide variety of molecules can also interact with laponite through a variety of methods, depending on their size, the pH of the medium, and their electrostatic properties. These interactions could occur at different regions of the laponite crystals: interparticle, interlayer, or surface or edge regions [103].

Laponite is suitable for a wide range of applications since it is a rheological modifier and a film-forming agent. Laponite XLG (gel-forming) is a suitable grade for biomedical purposes since it contains low amounts of heavy metals and hence it is less toxic. The degradation of laponite occurs under acidic conditions and causes the release of aqueous silica (Si(OH)_4_), sodium, magnesium, and lithium ions into the medium with low levels of toxicity [10,103].

#### 5.2.1. Drug Delivery Applications

Intravitreal injection is a commonly used administration route in ophthalmology to maintain therapeutic drug levels near the neuroretina. However, reinjections could cause vision-threatening adverse effects. In order to treat glaucoma for a prolonged length using intravitreal injection, a brimonidine–laponite nanocomposite was developed and an in vivo study suggested controlled drug release and a therapeutic effect for up to 6 months [104].

A dexamethasone–laponite nanocomposite was designed and the interaction between the non-ionic drug and laponite was obtained mostly by hydrogen bonding involving hydroxyl and carbonyl groups. The system was able to control the drug release, and preliminary preclinical in vivo testing seemed promising for its use as an intraocular drug release system [105].

Gellan gum (GG), a natural polysaccharide, was used to prepare spherical porous beads crosslinked with Ca^2+^ ions to be used as sustained drug delivery systems for oral administration of two model drugs, theophylline and cyanocobalamin, with different molecular weights. The presence of laponite lowered the swelling degree of the beads, increased the drug entrapment efficiency, and slowed down the release kinetics of both drugs in the gastric environment [106].

Hyaluronic acid, a natural polysaccharide able to bind to CD44, was conjugated onto laponite nanodisks. The expected goal was the targeted delivery of doxorubicin to CD44-overexpressed cancer cells. The nanocomposite had adequate in vitro biocompatibility, and specific anti-tumor effects on CD44-overexpressed HeLa cells, which selectively internalized the system by endocytosis [107].

A hydrogel drug delivery system based on laponite was developed by freezing/thawing using a poly(vinyl alcohol) matrix and loaded with rifampicin (Rif), chosen as an anti-COVID-19 repurposed drug. In silico and in vitro molecular docking simulations of the drug’s ability to inhibit the SARS-CoV-2 target protein 3-chymotrypsin-like protease were performed, in addition to experiments [108].

#### 5.2.2. Tissue Engineering Applications

Laponite has been mainly proposed in bone regeneration applications such as coating, scaffolds, bioink, and hydrogel.

An electrophoretic deposition approach was used to manufacture chitosan–laponite nanocomposite coatings with bone regeneration potential and regulated drug release ability. The intercalation of the polymer and drug macromolecules into silicate galleries allows for a controlled release of vancomycin, a glycopeptide drug [109].

Freeze-drying was also used to prepare a composite scaffold made of gelatin, laponite nanoparticles, and carboxymethyl chitosan for bone tissue regeneration. The addition of laponite induced an increase in cell proliferation and osteogenic differentiation but caused an inhibitory effect at higher concentrations [110].

In another work, a silk fibroin–laponite nanocomposite was loaded into fibrous membranes by means of electrospinning. Results demonstrated that the nanocomposite improved tensile strength, inducing the mineralization of bone apatite in a preclinical model [111].

Moreover, extrusion-bioprinted fibers produced from a nanocomposite bioink of laponite–gelatin methacryloyl showed interconnected porosity and controlled release of the loaded growth factors. The scaffolds also proved to enhance ex vivo vasculogenesis, as well as osteogenic differentiation, as demonstrated by the formation of mineralized nodules [112].

Injectable hybrid hydrogel microspheres based on RGD-alginate/laponite were developed and seeded with human dental pulp stem cells and enriched with vascular endothelial growth factor. The degradation rate of the microspheres in vitro was conditioned by the laponite content. Moreover, the systems were able to release bioactive vascular endothelial growth factor continuously for 28 days. Furthermore, the microspheres largely stimulated the regeneration of pulp-like tissues as well as the development of new micro-vessels in vivo in a preclinical model [113].

In another study, a composite of a silylated hydroxypropylmethyl cellulose hydrogel was developed and strengthened using laponite. The mixing process resulted in the formation of a hybrid interpenetrating network, which improved the mechanical characteristics of the hydrogel. In vitro studies revealed no toxic effects on human adipose stem cells, and the systems were also tested in vivo with a subcutaneous pocket implant in nude mice. The histological investigation proved the production of cartilage-like tissue with an extracellular matrix made of glycosaminoglycans and collagen [114].

Laponite was also used for the optimization of cardiac-inspired injectable hydrogels prepared using electroactive gold. The inorganic materials were loaded into a cardiac extracellular matrix to improve cardiomyocyte functional and biological characteristics, such as electrical conductivity. Cardiomyocyte cell survival and immunostaining investigations showed that the Au-loaded laponite hydrogel increased cell viability [115].

Another application involved the production of nano-laponite/polylactic acid–glycolic acid copolymer fibrous scaffolds able to replicate the human urethra tissue microenvironment. The microstructure of the fabricated scaffold was similar to that of the natural extracellular matrix. In particular, the scaffold containing laponite possessed the best mechanical properties with a strong elastic behavior. Moreover, the scaffold containing laponite also exhibited the best results for cell growth and proliferation when tested on HUVECs. The scaffolds therefore presented suitable characteristics to be used as urethral repair materials [116].

### 5.3. Layered Double Hydroxides

Layered double hydroxides (LDH) are a class of clays present in nature or easily synthesized at a cheap cost. Most LDHs have a structure comparable to the naturally occurring mineral hydrotalcite (Mg_6_Al_2_(OH)_16_CO_3_·4H_2_O) [117,118].

**Figure 12:**
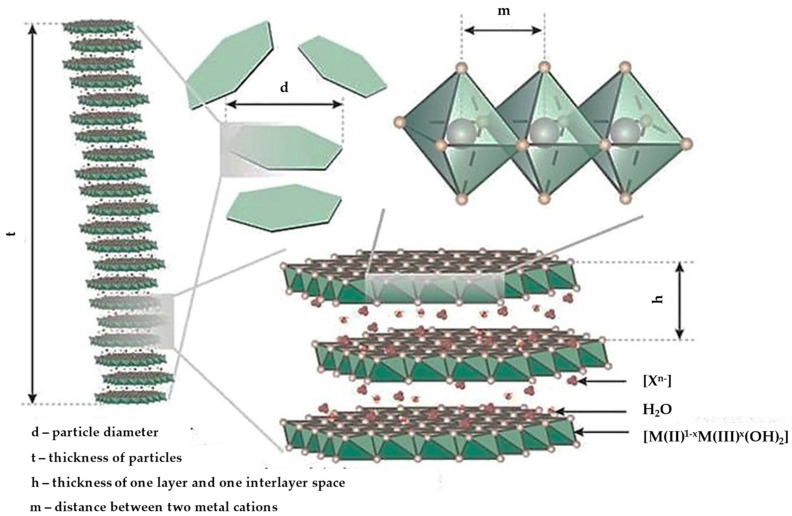
Schematic representation of LDH structure. Reproduced with permission from [120], CC BY 4.0.

**Figure 1:**
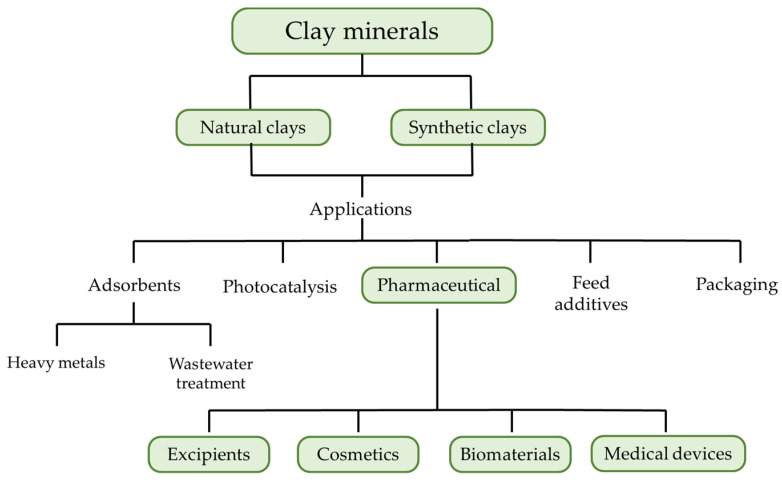
Common applications of clay minerals.

**Figure 2:**
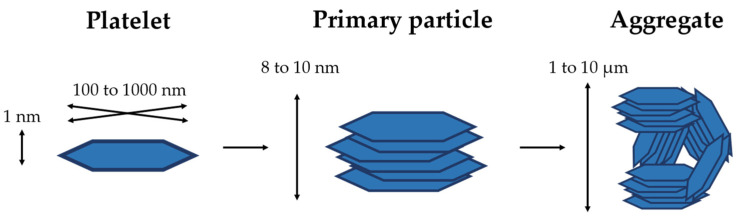
Scheme of the organization of the clay layers.

**Figure 3:**
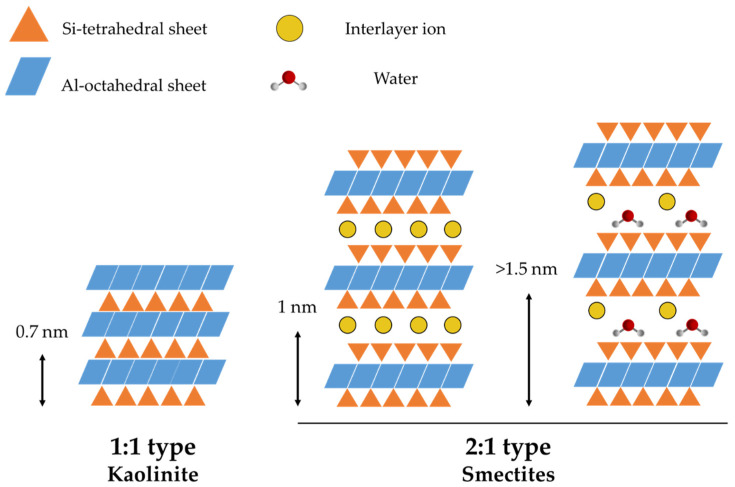
Various types of clay architectures and their unit cells. The influence of swelling on the interlayer distance is evidenced.

**Figure 4:**
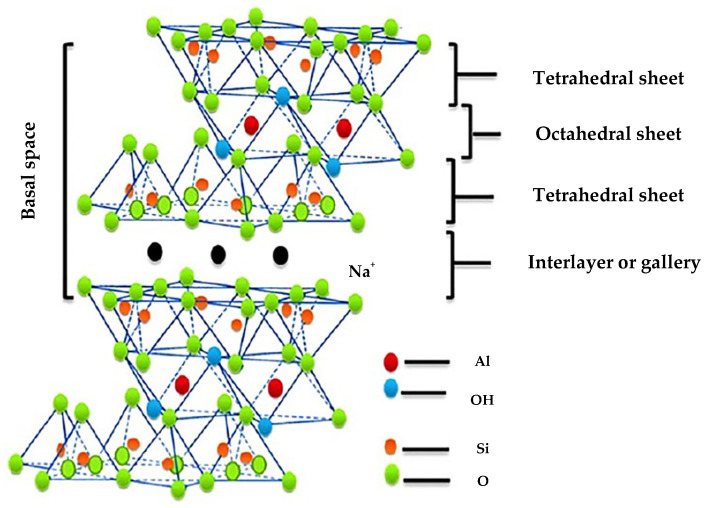
Schematic structure of montmorillonite. Adapted with permission from [30]. Copyright (2016) American Chemical Society.

**Figure 5:**
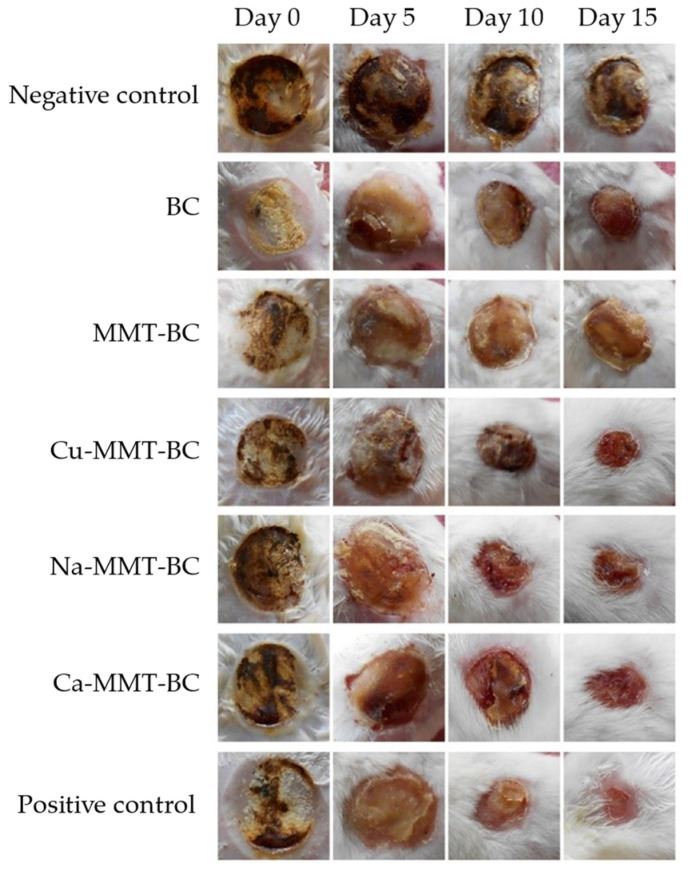
Representative wound photographs of Cu-MMT-BC, Na-MMT-BC, and Ca-MMT-BC treated group, negative control, BC, MMT-BC group, and positive control group as bandages for burn wounds during course of treatment. Adapted with permission from [44].

**Figure 6:**
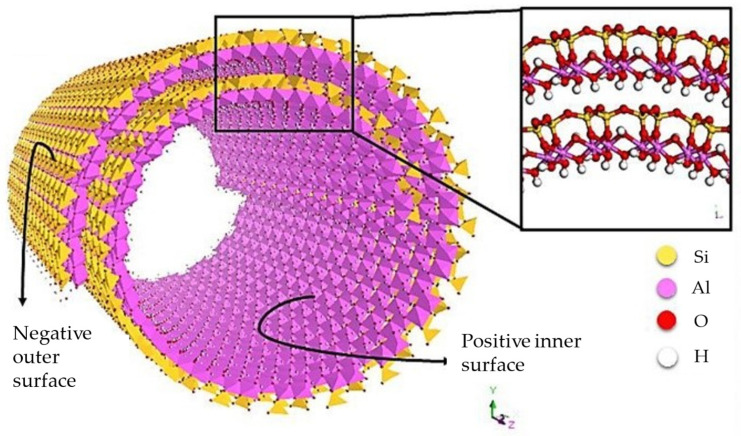
Structure of a halloysite nanotube. Adapted with permission from [48].

**Figure 7:**
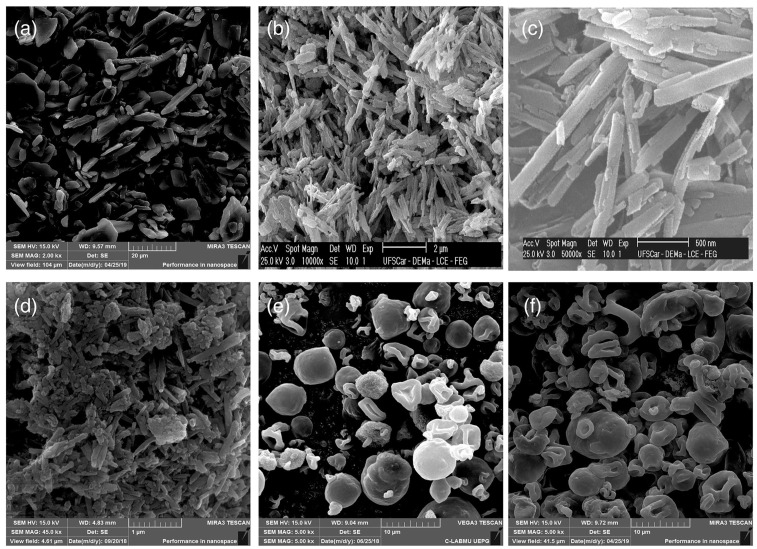
SEM images of (**a**) naproxen; (**b**) unloaded HNT (10 kx); (**c**) unloaded HNT (50  kx); (**d**) naproxen-loaded HNT; (**e**) nanocomposite (HNT:EC:HPMC 1:1:2); (**f**) nanocomposite (HNT:EC:HPMC 1:2:1). Reproduced with permission from [53].

**Figure 8:**
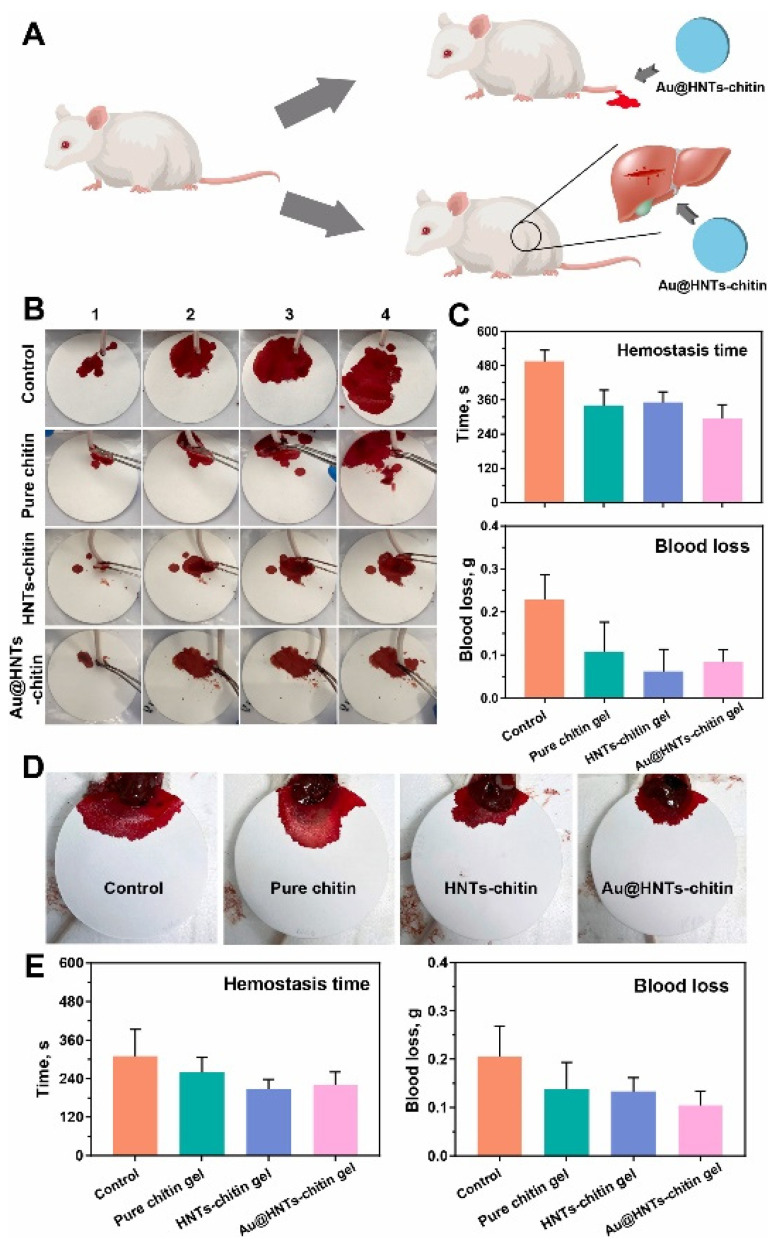
(**A**) Schematic representation of the mouse liver and tail hemorrhage model. (**B**) Photographs of tail hemorrhages in mice at different time periods. (**C**) Histogram of tail hemostasis time and blood loss in different groups of mice. (**D**) Photograph of a mouse liver with hemorrhage at the moment when the bleeding finally stopped. (**E**) Histogram of hemostasis time and blood loss in the liver of mice in different groups. Reproduced with permission from [65] CC BY-NC-ND 4.0.

**Figure 9:**
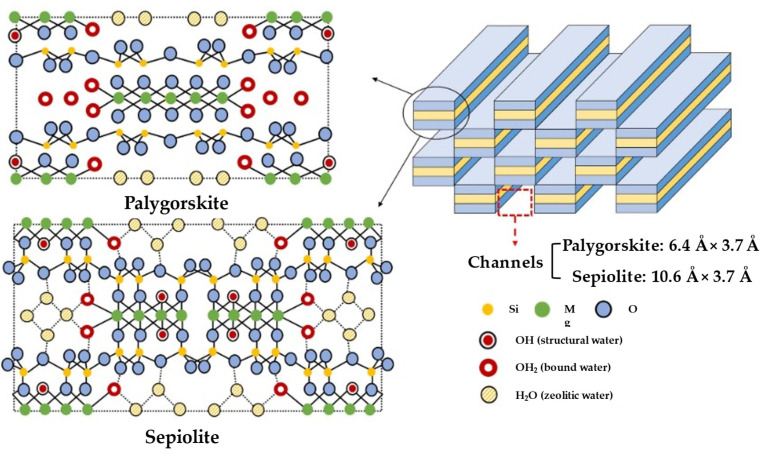
Structure of palygorskite and sepiolite clays. Adapted with permission from [68].

**Figure 10:**
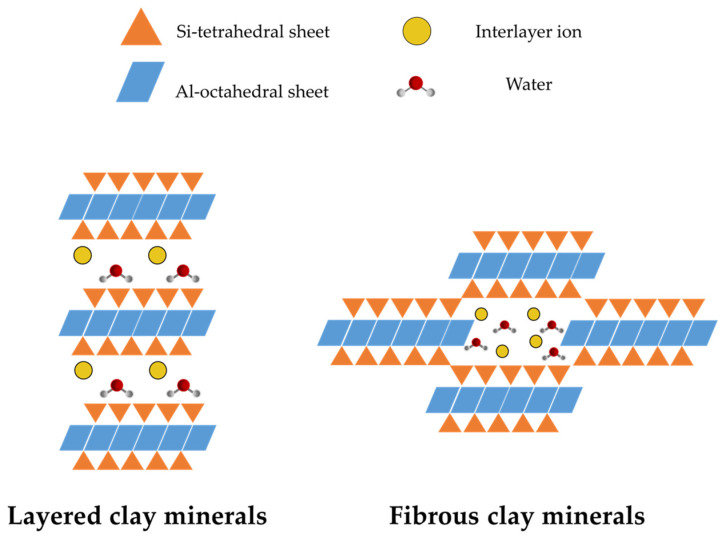
Schematic representation of the structural differences between layered and fibrous clays.

**Figure 11:**
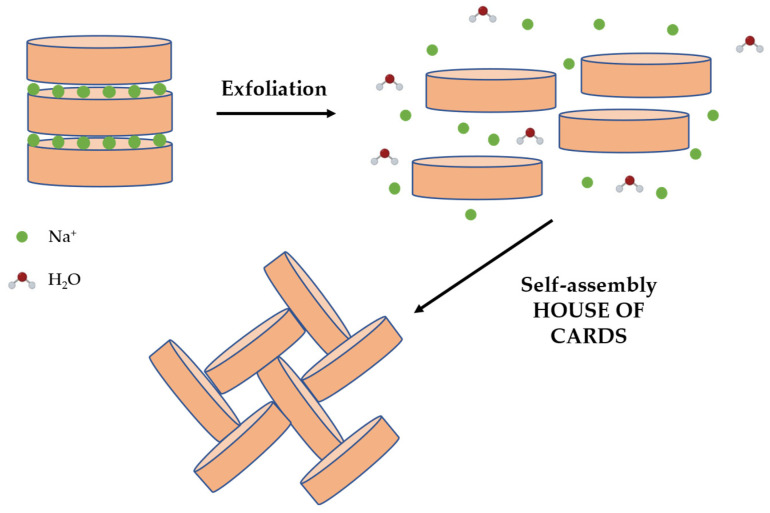
Schematic representation of exfoliation of laponite crystals in the presence of water. The single layers interact with each other forming the so-called “house of cards” structure, generating self-assembled gels.

LDHs are anionic lamellar compounds made of positively charged brucite-like layers with charge-compensating anions and water molecules in the interlayer region. Metal cations fill the centers of the octahedra, linked by hydroxide ions placed on their vertices, to form infinite two-dimensional sheets (Figure 12).

The generic formula for LDHs is [M^2+^_1−x_M^3+^_x_(OH)_2_][A^n−^]_x/n_·zH_2_O, where M^2+^ represents divalent cations such as Mg^2+^, Zn^2+^, or Ni^2+^, and M^3+^ represents trivalent cations such as Al^3+^, Ga^3+^, Fe^3+^, or Mn^3+^. A^n−^ is a non-framework charge compensating inorganic or organic anions, e.g., CO_3_^2−^, NO_3_^−^, Cl^−^, SO_4_^2−^, or RCO^2−^, and x is the mole fraction of M^3+^. The M^2+^ and M^3+^ cations deeply impact the charge density of the LDH sheets. This has implications for a number of physico–chemical properties such as reactivity, bonding, orientation, and mobility of interacting chemical molecules [119].

Several easy synthetic strategies can be employed to obtain LDH structure, such as the coprecipitation, hydrothermal, and ion-exchange methods. This allows for tailoring of the chemical composition of the structure and the intercalated molecules [6].

LDHs have several advantages, including a high surface-to-volume ratio, high hydrophilicity, strong chemical stability, pH-dependent solubility, and biocompatibility [121]. Due to their high mechanical strength, they are able to improve the mechanical properties of a polymer matrix and are used as a film-coating material for implant devices. The interaction with the positively charged layers determines a high anion exchange capacity leading to the release of the intercalated drug over time in a specific pH range. This mechanism decreases the clay’s positive charge, maintaining adequate levels for cellular absorption. A positive surface charge, in fact, causes the electrostatic interaction of LDH to the cell surface and promotes high membrane penetration, enhancing the transport of drugs or other macromolecules into different cell types. LDHs are quickly eliminated from the body and do not accumulate in other organs, demonstrating their biodegradability. The LDHs and the metal ions used to produce them are non-toxic compared to other nanomaterials such as iron oxide, silica, and carbon nanotubes. In terms of membrane damage, inflammation, and cell proliferation, LDHs with a size range of 100–200 nm proved to be safe [122].

The first biomedical application of LDHs was their use as active ingredients in antacids and anti-pepsin agents. In particular, MgAl-LDH is significantly able to raise the pH of the stomach and relieve stomach pain, indigestion, heartburn, and other symptoms associated with hyperacidity [37]. For other biomedical purposes, LDHs’ solubility is extremely interesting, and they are soluble at pH values below 4 and therefore able to release any intercalated drug for absorption in an acidic environment by solubilization. Moreover, LDHs possess viscoelastic characteristics comparable to those of gastric mucin. For this reason, these compounds have been used also in drug delivery systems, particularly to enhance the drug solubility and the gastroprotection of anti-inflammatory drugs [11].

#### 5.3.1. Drug Delivery Applications

LDHs are gaining interest as synthetic clays for drug delivery due to their anion exchange properties.

**Figure 13:**
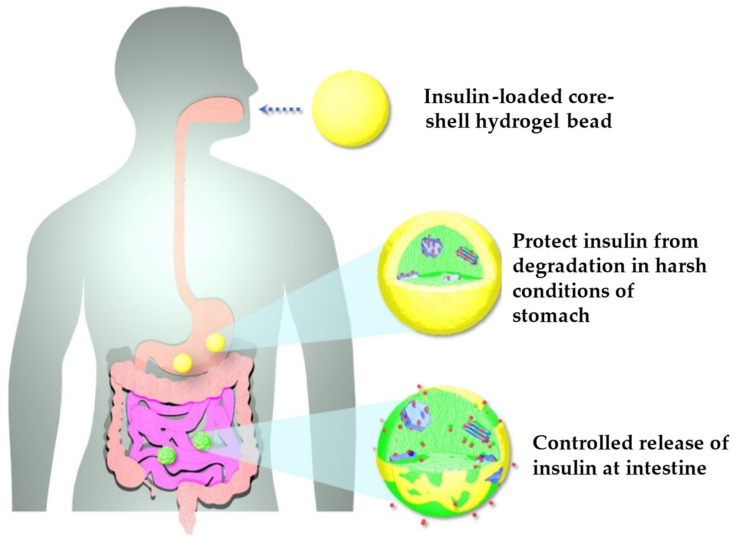
Schematic illustration of oral delivery of insulin using hydrogel beads. Adapted with permission from [124].

MgAl LDH has received great attention as a carrier for different types of drugs such as methotrexate [123], insulin [124], atorvastatin [125], and flurbiprofen [126]. Co-precipitation is described as the prevalent manufacturing method. In all cases, the intercalation of the various drugs was obtained, and the drug release was controlled over time. In the specific case of insulin LDH/insulin complexes, they were first coated with chitosan and subsequently with Ca alginate to produce core–shell hydrogel beads (Figure 13) that were able to further protect the drug against the gastro-intestinal environment, which is particularly challenging for its chemical stability [124].

MgAl LDH was also considered a carrier in local ophthalmic delivery. Hyaluronic acid-coated flurbiprofen-LDH (HA-FB-LDH) was prepared using co-precipitation and the stirring–ultrasonication method and allowed to achieve extended corneal residence time and to boost corneal permeability without irritation [126].

ZnAl LDH is another well-characterized LDH that was also proposed as a carrier to control nicotinic acid (vitamin B3) [127] and nisin peptide release [128]. The nanocomposites were prepared via co-precipitation and the drugs were intercalated. They achieved drug stability and sustained drug release.

#### 5.3.2. Tissue Engineering Applications

LDHs have been widely explored in tissue engineering since they are able to enhance the mechanical properties of polymeric matrixes and enhance cell adhesion and proliferation [129].

Electrospinning has been largely used and MgAl LDH was loaded in PCL/gelatin nanofibrous scaffolds for nerve tissue engineering [130] or in PCL nanofibers for bone tissue engineering [131]. The presence of LDH increased the mechanical strength and elongation at the break of the scaffold while decreasing the degradation rate. Moreover, the scaffolds improved attachment, proliferation, and normal cell behavior, including extracellular matrix production. The Mg-Al LDH loaded with enoxacin was also included in a polyurethane-polyvinyl alcohol hydrogel and, even in this form, LDH proved to control drug release and enhance chronic wound healing [132].

PCL electrospun scaffolds were also doped with CaAl LDH loaded with vitamin D3 to be used in bone tissue engineering. The presence of Ca^2+^ and vitamin D3 in micelles further enhanced the effectiveness of LDH in supporting osteoblast-like cell proliferation [133].

LDHs made of three cations were also tested and, in particular, MgSrFe-LDH associated with chitosan and Ag^+^ ions was prepared. This was freeze-dried to form a sponge-like structure intended as a bone scaffold, with a three-dimensional interconnected structure and pore sizes ranging from 100 to 300 nm. The systems assured an optimal proliferation and differentiation of the human bone marrow-derived mesenchymal stem cells together with antibacterial and antibiofilm properties [134].

LDHs based on different cations, in particular Mg^2+^, Zn^2+^, Fe^3+^, and Al^3+^, were synthesized and nanocomposites with polyether polyamide were manufactured. Naproxen was intercalated and a controlled release was obtained. The systems were biocompatible with fibroblasts in vitro and demonstrated enhanced wound healing [135].

Ciprofloxacin was intercalated in ZnAl LDH and entrapped in hyaluronan films by solvent casting. These showed antibacterial activity against *S. aureus* [136].

### 5.4. Zeolites

**Figure 14:**
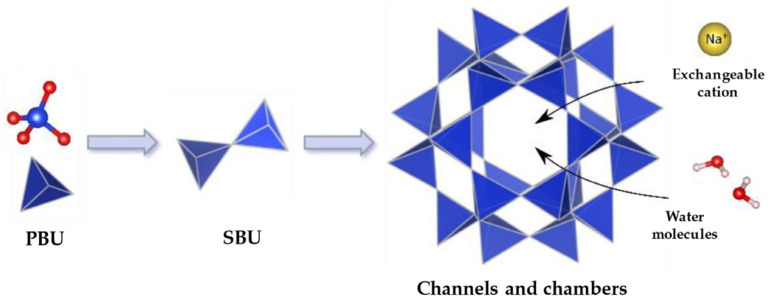
Scheme of the zeolite structure. Adapted with permission from [138], CC BY 4.0.

Zeolites are hydrated tectosilicates that can be found in nature as minerals as well as synthesized. Zeolites consist of tetrahedra made of Si and Al, which serve as the primary building units (PBU). Through bridging oxygen atoms, these tetrahedra are joined into a corner-sharing network (secondary building units, SBU). These units form frameworks with a consistent distribution of molecular-sized holes and cavities ranging in size from 4 to 12 Å (Figure 14). The presence of Al^3+^ could provide the zeolite tetrahedra with a negative charge, which could be compensated by exchangeable extra-framework cations, typically alkali or alkali-earth metals [137].

The general formula for zeolites is M^+^_x_ M^2+^_y_[Al_(x+2y)_Si_n−(x+2y)_O_2n_]·mH_2_O, where M^+^ and M^2+^ represent extra-framework alkali metals and alkali-earth metals, respectively. The values x and y are linked to the molar concentrations of Al and Si in the zeolite framework, and n is the metal valence. Some zeolites can also be purely siliceous [139]. In addition, zeolite post-synthesis modifications could change the Al or Si composition of the framework, leading to increased hydrophobicity and hydrothermal stability [140].

The International Zeolites Association has identified over 200 framework types and over 50 known mineral families of zeolites. The framework classification is based on structural topology, whilst the family classifications depend on mineral composition. Each framework type has its own three-letter code. In most cases, the codes are taken from the names of the material type. Different characteristics can be considered for the classification of zeolites:

(A) Pore size (depending on the interparticle arrangement of zeolites channels and cavities):
  - Microporous, having pore diameter <2 nm;
  - Mesoporous, having pore diameter 2–50 nm;
  - Macroporous, having pore diameter >50 nm;
(B) Acidity (depending on the Si/Al ratio):
  - Low silica (Si/Al ratio 1.0–1.5) (Zeolite X);
  - Intermediate silica (Si/Al ratio 2.0–3.0) (Zeolite Y);
  - High silica (Si/Al ratio 10–250) (Zeolite ZSM-5).

The structure features of the zeolites provide them with interesting properties, including:

  - Low density and a large volume of free spaces;
  - High degree of hydration;
  - High degree of crystallinity;
  - High internal surface area, causing the adsorption of molecules and ions;
  - Ion exchange ability;
  - Catalytic ability [138].

Zeolites are widely used since they were designated as ‘non-toxic’ by the International Agency for Research on Cancer (IARC) and “safe for human consumption” by the Food and Drug Administration (FDA). The European Food Safety Authority (EFSA) has authorized the clinoptilolite zeolite type as a safe food and feed ingredient. This results in their widespread use in different fields, such as agronomy, as dietary supplements in animal diets, as insecticides and pesticides in plant protection, and as anticarcinogenic compounds, and they have seen successful use in healing cuts and wounds. Due to their large surface area and porosity, zeolites are used in many technological applications, including as molecular sieves in catalysis and as adsorbents. These materials are also more appealing and frequently utilized for biomedical applications, such as wound healing, due to their biocompatibility, low toxicity, and antibacterial activity. Furthermore, the porosity of the zeolite framework favors the loading and the release of drugs, making it acceptable for the production of delivery systems [141,142].

#### 5.4.1. Drug Delivery Applications

The porosity and surface area of zeolites make them interesting materials for the loading of active ingredients. Different drug types were adsorbed into the channel of zeolites. For example, a faujasite zeolite was loaded with isoniazid for antituberculosis treatment via the oral route [143] and with 5-fluorouracil for oral cancer therapy [144]. The ionization degree of the drug deeply influenced the drug loading and its release giving a pH-dependent release. X- and Y-type zinc zeolites were loaded with 6-mercaptopurine via coordination between zinc cations and the sulfur and nitrogen atoms. This allowed for the control of the drug’s release via the oral route [145].

#### 5.4.2. Tissue Engineering Applications

As all the inorganic materials as well as the zeolites provide mechanical resistance, they are able to confer antibacterial activity to the polymers.

**Figure 15:**
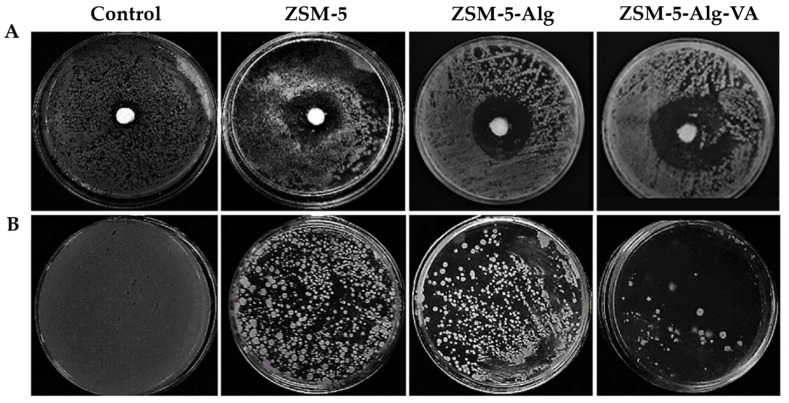
Antibacterial activity of ZSM-5 scaffolds against *S. aureus*: (**A**) inhibition zones of the ZSM-5 scaffolds with Alg and VA-loaded Alg coating, (**B**) plate count technique CFU results. Adapted with permission from [146], CC BY 4.0.

Nanocomposites based on different types of zeolites and polymers were proposed in the literature and alginate [146], gelatin/agarose [147], pectin [148], hyaluronic acid [149], ethylcellulose/polyvynil pyrrolidone [150], and Pluronic F127/chitosan [151] were considered. In particular, a bone scaffold based on alginate and ZSM-5 and loaded with vancomycin demonstrated superior antibacterial activity against *S. aureus* and excellent osteoblast adhesion and calcium deposition, as shown in Figure 15 [146].

Analogously, a hydrogel based on gelatin/agarose mixed with a pomegranate-peel-extract-laden zeolite was designed for skin healing [147]. A film based on a pectin/copper zeolite was developed for wound therapy and proved to be effective in vitro in fibroblast cell culture and in vivo in rats [148]. Nanoparticles based on a hyaluronic-acid-modified zeolite loaded with imidazole and curcumin for the treatment of burns were manufactured to control the abnormal extracellular matrix deposition in the remodeling stage. This possesses a remarkable antibacterial activity against *P. aeruginosa*, *S. aureus,* and *E. coli* and simultaneously was able to enhance fibroblast proliferation and angiogenesis [149].

Electrospun ethyl cellulose/polyvinylpyrrolidone scaffold-doped zeolites ZIF 8 were able to promote fibroblast proliferation and release Zn^2+^ ions with antimicrobial activity [150]. A thermosensitive hydrogel made of Pluronic F127 and chitosan was loaded with cerium-doped LTA zeolite nanoparticles. These demonstrated the capability to fasten the transition from inflammation to proliferation, as well as remodeling capabilities. Ce^3+^/Ce^4+^ oxidation conferred antioxidant properties to the system by reducing ROS formation. This proved to increase epithelialization, production of granulation tissue, neovascularization, and collagen remodeling at the wound site [151].

The drug delivery and tissue engineering applications of zeolites, together with all the other natural and synthetic clays mentioned above, are reported in Table 2.

## 6. Conclusions

Clay minerals are relevant materials for use in the pharmaceutical and biomedical fields, as they are well-known pharmaceutical excipients and possess therapeutic properties. They are also important in drug delivery and tissue engineering. The clays possess unique physico–chemical properties (3D structure and chemical composition, morphology, porosity, high surface area, and ion exchange ability) which make them interesting for the development of therapeutic platforms. Most importantly, they are non-toxic and biocompatible materials that are traditionally used as disintegrants or diluents in solid dosage forms or as emulsifying or thixotropic agents in semisolids. Currently, nanocomposites based on a drug (and eventually a polymer) are under development as drug carriers to control drug release and to increase poorly soluble drug availability. Moreover, the inclusion of clay minerals in a polymeric matrix confers superior mechanical and thermal properties to the resulting nanocomposites and is also able to support cell adhesion and proliferation in tissue regeneration, depending on the polymer type.

Clay minerals in pharmaceutical and biomedical fields are versatile and widely studied, as deeply described in this review. Despite the plentiful literature on this topic, particular attention should be paid to the degradation rate and the risk of accumulation in the body since a few materials are relatively insoluble and could last longer if implanted. On this matter, LDH, being soluble in acidic environments, are more promising synthetic materials that could have a higher purity degree and are devoid of toxic impurities. Although further studies are certainly needed to reach the market as a drug carrier in nanocomposites and as a component in 3D structure for tissue engineering, the research activities in these fields are many and lead to hope for a rapid evolution towards the clinic.

## Figures and Tables

**Table 1:** Main differences between layered and fibrous clays. Adapted with permission from [69].

Layered Clays	Fibrous Clays
1-nanodimensional particle	2-nanodimensional particle
High charge density	Low charge density
High CEC	Low CEC
Low density silanol groups	High density silanol groups
High internal surface area	Low internal surface area
Particles in layer stacks	Particles in bundles
Swelling clay	Non-swelling and non-exfoliable clay

**Table 2:** Applications of natural and synthetic clays for drug delivery (DD) and tissue engineering (TE) applications and major results from in vitro/in vivo studies.

Clay Type	Polymer	Drug	Targeted Application	In Vitro/In Vivo Studies	Major Results of In Vitro/In Vivo Studies	Ref.
MMT	PLGA	Dexamethasone	Oral DD	-	-	[36]
	ι-carrageenan/gelatin	Ciprofloxacin	Oral DD	-	-	[37]
	Chitosan	Oxytetracycline	Oral DD	Toxicity and permeation on Caco-2 cells	All the samples showed good biocompatibility at the studied concentrations. Moreover, the OXT loading into the nanocarriers did not cause cytotoxic effect on Caco-2 cell line	[38]
	Sodium alginate	Curcumin	Oral DD	-	-	[39]
	Eudragit^®^PO	Betaloxol hydrochloride	Ophthalmic DD	CAM-TBS irritation assay	The MMT-betaloxol system was well tolerated but showed slight irritation due to the intrinsic irritancy of the active pharmaceutical ingredient (API) adsorbed on the surface of nanoparticles.	[40]
	Polyacrilamide/NaCMC	Methylene blue (model)	Vaginal DD	Toxicity on human skin fibroblasts (HSF 1184)	The hydrogels proved to be biocompatible. A slight decrease in cell viability was observed as the concentration of MMT was increased. This may be due to the blockage of most of the channels on cell membranes.	[41]
	Polyurethane	Chlorhexidine acetate	Skin TE	-	-	[42]
	Chitosan	Norfloxacin	Skin TE	Toxicity on normal human dermal fibroblasts (NHDF)	The scaffolds supported cell viability and proliferation after 6 days. The loading of norfloxacin caused a significant decrease in cell viability which could be completely attributed to the drug and not to the scaffold.	[43]
	Bacterial cellulose	-	Skin TE	Evaluation of wound healing activity on mice model	The wound healing activity in animals treated with nanocomposites was increased, together with tissue regeneration, vascularization and re-epithelialization	[44]
	Chitosan/gelatin	-	Bone TE	-	-	[45]
	Polyhydroxybutyrate	-	Bone TE	Toxicity on lymphocites	The addition of modified MMT increased cell viability compared to the polymer alone, with the greatest increase showed with the addition of 7% MMT.	[46]
	Silk fibroin	-	Bone/Cartilage TE	Toxicity on BMSCs and chondrocytes; Osteochondral regeneration on white rabbits	The MMT-reinforced SF hydrogel showed good cytocompatibility, as well as the bioactivities to induce osteogenesis. In vivo, SF-MMT treated groups presented a better cartilage regeneration, a more mature hyaline cartilage formation, an enhanced and integrated subchondral bone regeneration, and a more vertical-aligned arrangement compared, indicating a more physiological structure of regenerative cartilage	[47]
HNT	-	Isoniazid	Oral DD	Toxicity and permeability on Caco-2	INH-loaded HNT showed good biocompatibility at the concentrations studied (up to 1233 μg/mL), with improved cell proliferation. Permeability tests showed that INH transport across cellular membranes was greatly enhanced and the drug encapsulated into nanohybrid was effectively internalized by the cells	[52]
	EC/HPMC	Naproxen	Oral DD	-	-	[53]
	Chitosan	Curcumin	Targeted DD (breast cancer)	Toxicity on MCF-7	After 24 h of incubation, the HNT-treated cells recorded cell viability comparable with the control group (94%). The combination of curcumin and HNT increases cell viability compared to the pure drug.	[54]
	-	Rhodamin/ionomycine (model)	-	Toxicity and Ca2+ response on BMVECs	HNT-ionomycin nanocapsules showed a gradual influx of Ca^2+^ and higher delivery of ionomycin in the cells, which was much higher compared to HNT alone.	[55]
	-	Clotrimazol	Vaginal DD	-	-	[56]
	PLA	-	Skin TE	Toxicity on fibroblasts	Cell viability of the PLA/HNT scaffolds was improved compared with neat PLA foams. The highest cell viability ratio was obtained in scaffolds containing 3% HNT.	[61]
	Chitosan	-	Skin TE	Toxicity on fibroblasts; Wound healing activity on male rats	After 7 days of treatment, the histological analysis of threated samples allowed an early re-epithelialization process and an advanced degree of hemostasis and angiogenesis. Moreover, the presence of granulation tissue was observed.	[62]
	Chitosan/PVA	-	Skin TE	Toxicity and proliferation on fibroblasts	The biocompatibility of the nanocomposite samples containing HNT were higher than samples without HNT as well as control. Incorporation of HNT enhanced the cell attachment and proliferation on the samples.	[63]
	Dialdehyde corn starch/Gelatin/Bacterial nanocellulose	-	Skin TE	Toxicity and proliferation on NIH-3T3 fibroblasts	Cell viability in presence of films containing BC and HNT was increased, due to the electrostatically interaction of BC and HNT with growth factors and other ECM proteins, and to the capability of the scaffold to improve the hydrophilicity nature and protein absorption on its surface.	[64]
	Chitin	-	Skin TE	Toxicity on mouse fibroblast cell line L929; Wound healing ability in mice model	The HNT-chitin group had a wound healing effect with a reduced wound area compared to the control. It also showed has the highest collagen deposition density, which is similar to the control group. The hydrogel showed high hemostatic performance in mouse liver and tail bleeding, together with antibacterial activity.	[65]
	Sodium alginate/collagen	Clindamycin	Bone TE	Toxicity on Hs27 Human foreskin fibroblasts, WM793 human melanoma and human osteosarcoma (MG-63) cells	The cytocompatibility of materials containing HNT and their antibacterial effect on *S. aureus* was confirmed.	[66]
	PCL-PEG-PCL/Gelatin	-	Bone TE	Cell viability and proliferation on hDPSCs	The prepared composites were non-toxic even if a decrease in viability for scaffolds containing HNT of 6% was observed. The incorporation of HNT enhanced the surface volume of scaffolds and increased their roughness, providing superior interaction with cells.	[67]
LDH	-	Methotrexate	Oral DD	Toxicity HCT-116 cell line	After 48 h incubation, at IC50 concentration of methotrexate (MTX), LDH–MTX nanohybrid was found to inhibit 50% of the cells, which is an increased value compared to the control. These results clearly demonstrated higher efficacy of LDH–MTX nanohybrid compared to the pure MTX. As expected, pristine LDH has shown little cytotoxicity as the control on the proliferation (~95%).	[123]
	Alginate-Chitosan	Insulin	Oral DD	Biocompatibility CAM irritation assay	Core-shell hydrogel beads implanted chick embryo revealed good angiogenic response. Furthermore, the core–shell beads recovered from the chick embryo showed micro vascularity in the beads.	[124]
	-	Valsartan/Atorvastatin	Oral DD	-	-	[125]
	Hyaluronic acid	Flurbiprofen	Ophthalmic DD	In vivo pharmacokinetics and irritation study, ex vivo ocular distribution on white rabbits	Both in vivo ocular irritation test results and ocular pathological tissue section results illustrated that flurbiprofen-loaded LDH were not irritating to the eyes. The addition of hyaluronic acid could prolong the residence time of the drug in front of the cornea. In addition, bioavailability of flurbiprofen in the eyes could be improved.	[126]
	-	Nicotinic acid	Transdermic DD	-	-	[127]
	-	Nisin	-	-	-	[128]
	PCL/Gelatin	-	Nerve TE	Toxicity on human neuroblastoma SH-SY5Y cells	Cell migration and proliferation were enhanced in nanofibers containing LDH, as they led to more hydrophilicity and higher protein adsorption.	[130]
	PCL	-	Bone TE	Toxicity on human MG-63 osteoblast-like cells.	The LDH/PCL scaffold induced mineral deposition and osteogenic differentiation as evident by an increase in ALP activity in comparison with the pure PCL.	[131]
	Polyurethane/PVA	Enoxacin	Skin TE	-	-	[132]
	PCL	Vitamin D3	Bone TE	Toxicity, cell adhesion, proliferation on MG-63 cell line	The addition of vitamin D3-LDH nanohybrid showed a great influence on improving cells adhesion to the scaffold surface, their spreading and the attachment of cells to each other.	[133]
	Chitosan		Bone TE	Toxicity on hBMSC cells	The in vitro cell tests reveal that all the LDH composite scaffolds exhibit excellent cytocompatibility. The presence of Ag and Sr contribute to osteogenic activity and antibacterial property, respectively. The released Sr2+ ions enhance the ALP activity, promote the ECM mineralization, and increase the expression levels of osteogenic-related RUNX2 and BMP-2.	[134]
	PEBA	Naproxen	Skin TE	Toxicity on NHDF	The incorportation of LDH in PEBA displayed an improved biological response in comparison to the pristine polymer. The intercalation of naproxenate into Mg-LDH decreased the cytotoxicity of the drug in comparison with the pristine powder and its PEBA composite.	[135]
	Sodium hyaluronan	Cyprofloxacin	Skin TE	-	-	[136]
ZEO	-	Isoniazid	Oral DD	-	-	[143]
	-	5-fluorouracil	Oral DD	Toxicity on Caco-2 cells	The drug-loaded versions of zeolite showed significant cytotoxic towards a tumoral cell-line compared to the pristine zeolites. Notably, the presence of Al released from the zeolite did not improve the cytotoxic activity of the zeolite, contradicting the idea of the poisonous effect of Al.	[144]
	-	6-mercaptopurin	Oral DD	Toxicity on Mcf-7 cells	The tested materials containing 6-mercaptopurin released the drug and affected cancer cell viability, as opposed to unloaded zeolites. Two different zeolites were tested, with zeolite Y that released the drug (88%) more efficiently than zeolite X (78%) after 30 h incubation.	[145]
	PCL	Cisplatin	Bone TE	Toxicity on MG63 cells	the addition of 20% Zeol increased the in vitro bioactivity making it a suitable option for bone tissue engineering	[152]
	Alginate	Vancomycin	Bone TE	Toxicity on MG63 cells	After 7 days of culture, osteoblast cells were attached and proliferating, with a strong anibacterial effect given by the presence of vancomycin.	[146]
	Gelatin/Agarose	pomegranate peel extract	Skin TE	Toxicity on L929 mouse fibroblast cell line	The sample contaning 0.5% zeolite displayed a slightly higher cell proliferation compared to other samples. The zeolite addition up to 2% did not affect the hydrogel biocompatibility and cell proliferation.	[147]
	Pectin		Skin TE	Toxicity on NIH 3T3 fibroblasts; Wound healing on male rats	Pectin containing 1% of Cu-exchanged Faujasite exhibited excellent antibacterial activity and cell viability and aided in improving wound healing and re-epithelialization in Sprague Dawley rats.	[148]
	Hyaluronic acid	Curcumin	Skin TE	Toxicity on mouse fibroblasts L929; Wound healing on mice model	The application in wounds in vivo shows that the scaffold can promote the proliferation of fibroblasts and accelerate angiogenesis. Importantly, histological analysis showed normal collagen density compared to free curcumin and control groups.	[149]
	EC/PVP	-	Skin TE	Toxicity on NIH3T3 fibroblasts	Scaffolds expressed higher percentages of cell proliferation compared with the control group. Moreover, the proliferation percent reached its highest value after 5 days of culture. Lowering the MW of PVP led to better results in terms of cell viability.	[150]
	Pluronic F127/Chitosan	-	Skin TE	Toxicity on HUVECs; Wound healing on mice model	The scaffolds containing zeolites showed improved proliferation, migration and angiogenesis. Furthermore, in vivo studies confirmed the formation of granulation tissue, re-epithelialization and collagen remodeling at the wound site, thereby accelerating the healing process	[151]

## Data Availability

Not applicable.
